# The yield difference between wild-type cotton and transgenic cotton that expresses IPT depends on when water-deficit stress is applied

**DOI:** 10.1038/s41598-018-20944-7

**Published:** 2018-02-07

**Authors:** Xunlu Zhu, Li Sun, Sundaram Kuppu, Rongbin Hu, Neelam Mishra, Jennifer Smith, Nardana Esmaeili, Maheshika Herath, Michael A. Gore, Paxton Payton, Guoxin Shen, Hong Zhang

**Affiliations:** 1 Department of Biological Sciences, Texas Tech University, Lubbock, TX 79409 USA; 2 USDA-ARS, Arid-Land Agricultural Research Center, Maricopa, AZ 85239 USA; 3 USDA-ARS, Cropping Systems Research Laboratory, Lubbock, TX 79415 USA; 4 Zhejiang Academy of Agricultural Sciences, Hangzhou, Zhejiang Province China; 5 Present Address: Plant Breeding and Genetics Section, School of Integrative Plant Science, Cornell University, Ithaca, NY 14853 USA

## Abstract

Drought is the No. 1 factor that limits agricultural production in the world, thus, making crops more drought tolerant is a major goal in agriculture. Many genes with functions in abiotic stress tolerance were identified, and overexpression of these genes confers increased drought tolerance in transgenic plants. The isopentenyltransferase gene (*IPT)* that encodes a rate limiting enzyme in cytokinin biosynthesis is one of them. Interestingly, when *IPT*-transgenic cotton was field-tested at two different sites, Texas and Arizona, different results were obtained. To explain this phenomenon, reduced irrigation experiments with different timing in applying water deficit stress were conducted. It was found that the timing of water deficit stress is critical for *IPT*-transgenic cotton to display its yield advantage over control plants (i.e. wild-type and segregated non-transgenic plants). If water deficit stress occurs before flowering (vegetative phase), *IPT*-transgenic cotton would outperform control plants; however, if water deficit stress occurs at or after flowering (reproductive phase), there would not be a yield difference between *IPT*-transgenic and control cotton plants. This result suggests that an early induction of *IPT* expression (before first flowering) is needed in order to realize the benefits of *IPT*-expression in transgenic plants that face water-deficit stress later in development.

## Introduction

Fresh water shortage is the most critical factor that not only limits agricultural production, but also affects the stability of many countries. The most populous countries in the world such as China, India, Pakistan, and even United States of America, are facing a severe shortage in fresh water supply for agricultural production. According to United Nations’ projection, the world population will exceed 9 billion by 2050^1^. Consequently, the world food production would have to be increased by at least 70% to 100%^2^. Unfortunately, the agricultural lands that are suitable for food production will not likely be increased, instead they are steadily decreasing due to economic and urban development in many countries such as China and India. The production of more food with less land, especially with declining availability of fresh water, is a major challenge ahead. If food production is not increased in pace with the population growth, developing countries could experience famines and political instability as a direct result.

To increase agricultural production by 70% to 100% with existing land or less land, we must develop new cultivars that have higher yields or that minimize crop losses caused by pathogen infections and environmental stresses. It is estimated that environmental stresses cause between 30% to 50% crop losses worldwide annually^3^, among which drought is the No. 1 factor causing substantial crop losses^4^. If we can minimize drought-caused crop losses, we would be able to increase crop production immensely. Breeding for drought tolerant crop varieties has always been the top priority in agriculture for the last 100 years, and it was largely successful and contributed to the Green Revolution 50 years ago. However, due to the fast-growing population and depleting agricultural land and freshwater resources, depending on traditional breeding for more drought tolerant varieties could not afford to be the only solution to address this issue. Biotechnology based on recombinant DNA techniques was therefore adopted about 20 years ago, where many genes that have great potential in increasing drought tolerance in transgenic plants were discovered and tested in model plants. Some of these genes appear to be promising for crop improvement.

The first group of genes that showed potential in improving abiotic stress tolerance were those encoding transcriptional factors that activate gene expression in response to stimuli of environmental stresses^5,6^. For example, a group of genes in the families of DREBs or NACs encoding transcriptional factors that bind to the promoters of the downstream genes, are water deficit induced, thereby, activating downstream gene expression and enabling plants to acquire increased drought tolerance^7,8^. Another group of genes that showed great potential are the ones encoding functional enzymes or membrane-bound transporter proteins. For an example, the *AVP1* gene from Arabidopsis encodes a vacuolar membrane bound proton pump^9^. When *AVP1* was overexpressed in transgenic plants, it increased drought tolerance in Arabidopsis, tomato, peanut, and cotton^9–12^. The gene *OsSIZ1* from rice encodes a SUMO E3 ligase and overexpression of *OsSIZ1* could increase drought tolerance in transgenic plants substantially^13,14^.

Although many genes hold great promise in increasing drought tolerance in laboratory and greenhouse studies, so far very few have been commercialized, except in one case where Monsanto appeared to have released a drought tolerant maize line^15^. This maize line expresses a bacterial cold shock protein that aids in withholding water under drought conditions. It does not cause a penalty in yield while expressing this gene under normal growth conditions, and under mild drought conditions, it outperforms wild-type corn by maintaining 5–10% higher yields^15^. The success of the release of this drought-tolerant maize by Monsanto a few years back is not entirely clear. Yet, based on the limited information about this line, it can be assumed that unless the margin of the yield advantage is larger than 5–10% under drought conditions, it may be difficult to convince farmers to adopt this line for large scale cultivation, as they are required to pay a higher price to buy the transgenic seeds.

Why was there only limited success so far in genetically engineered crops for improved drought tolerance? One likely reason is that many genes did not perform well in field studies, despite producing excellent results from laboratory and greenhouse studies. An illustrative successful example is the *IPT* that encodes isopentenyltransferase, a rate limiting enzyme in cytokinin biosynthesis^16–18^. Rivero *et al*.^19^ showed that overexpression of *IPT*, under the control of a water deficit inducible promoter, conferred increased drought tolerance in transgenic tobacco plants. Similar results were obtained in rice, peanut, and cotton^12,20,21^. Interestingly, when *IPT*-transgenic cotton was tested in field conditions, different results were obtained; some showed that *IPT*-transgenic cotton produced higher yields than control plants, while others showed that no yield differences were found between *IPT*-transgenic cotton and control plants. To explain this phenomenon, we analyzed how *IPT*-transgenic cotton and control cotton would perform under different irrigation conditions. We found that the timing of water deficit stress in relation to the developmental stage of cotton plants is critical for *IPT*-transgenic cotton to display its advantage over control plants under drought conditions. This study provides a cautionary note on the use of *IPT* for improving drought tolerance in transgenic crops.

## Results

### *IPT*-expressing transgenic cotton performs better than wild-type and segregated non-transgenic cotton lines under dryland conditions in the field

**Figure 1:**
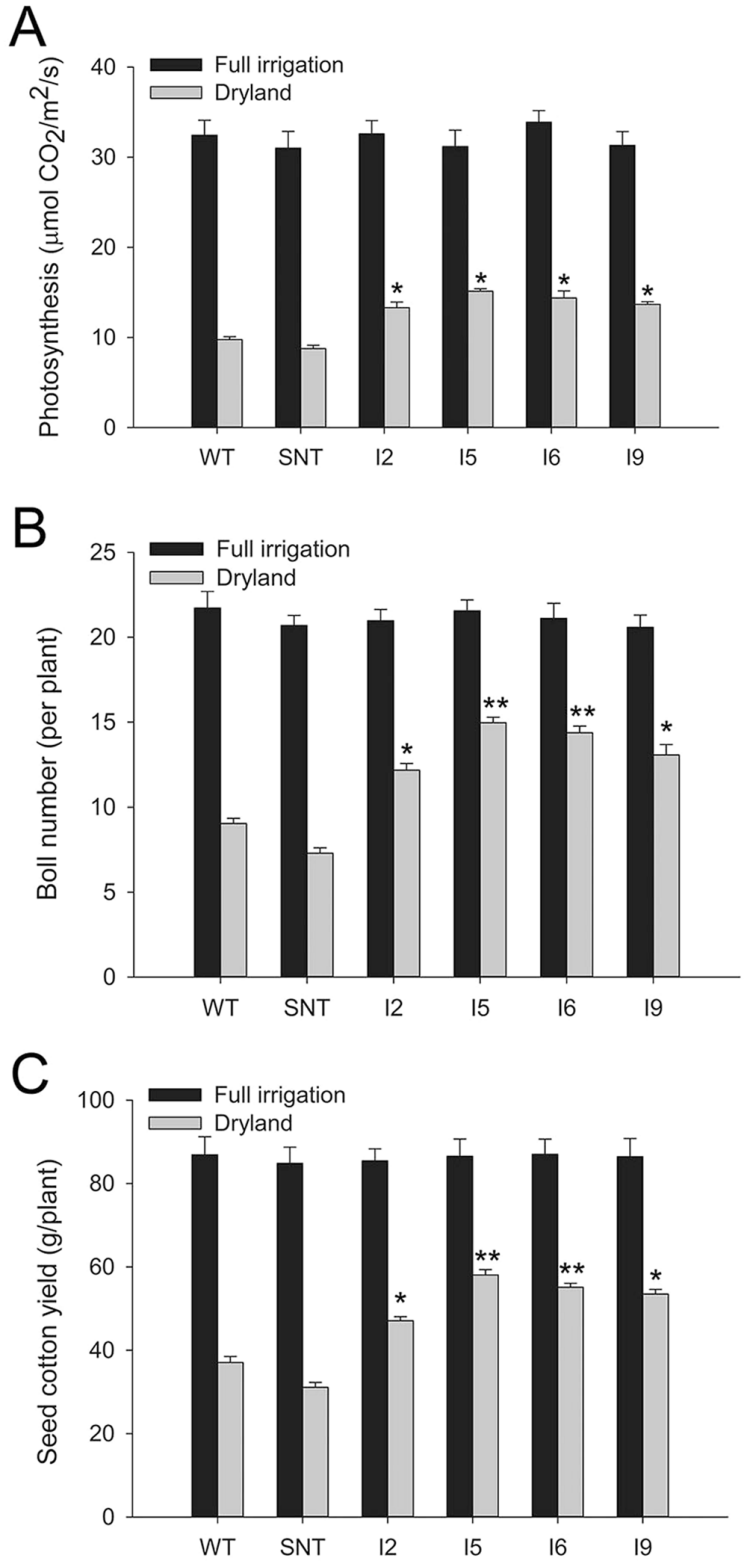
Photosynthetic rate, boll number, and seed cotton yield of cotton lines under full irrigation and dryland conditions in 2010. WT, wild-type cotton (Coker 312); SNT, segregated non-transgenic cotton; IPT2 to IPT9, four independent *IPT*-transgenic cotton lines. Black bar, full irrigation condition; grey bar, dryland condition. *Statistically significant at the < 0.05 level; **statistically significant at < 0.01 level.

**Figure 2:**
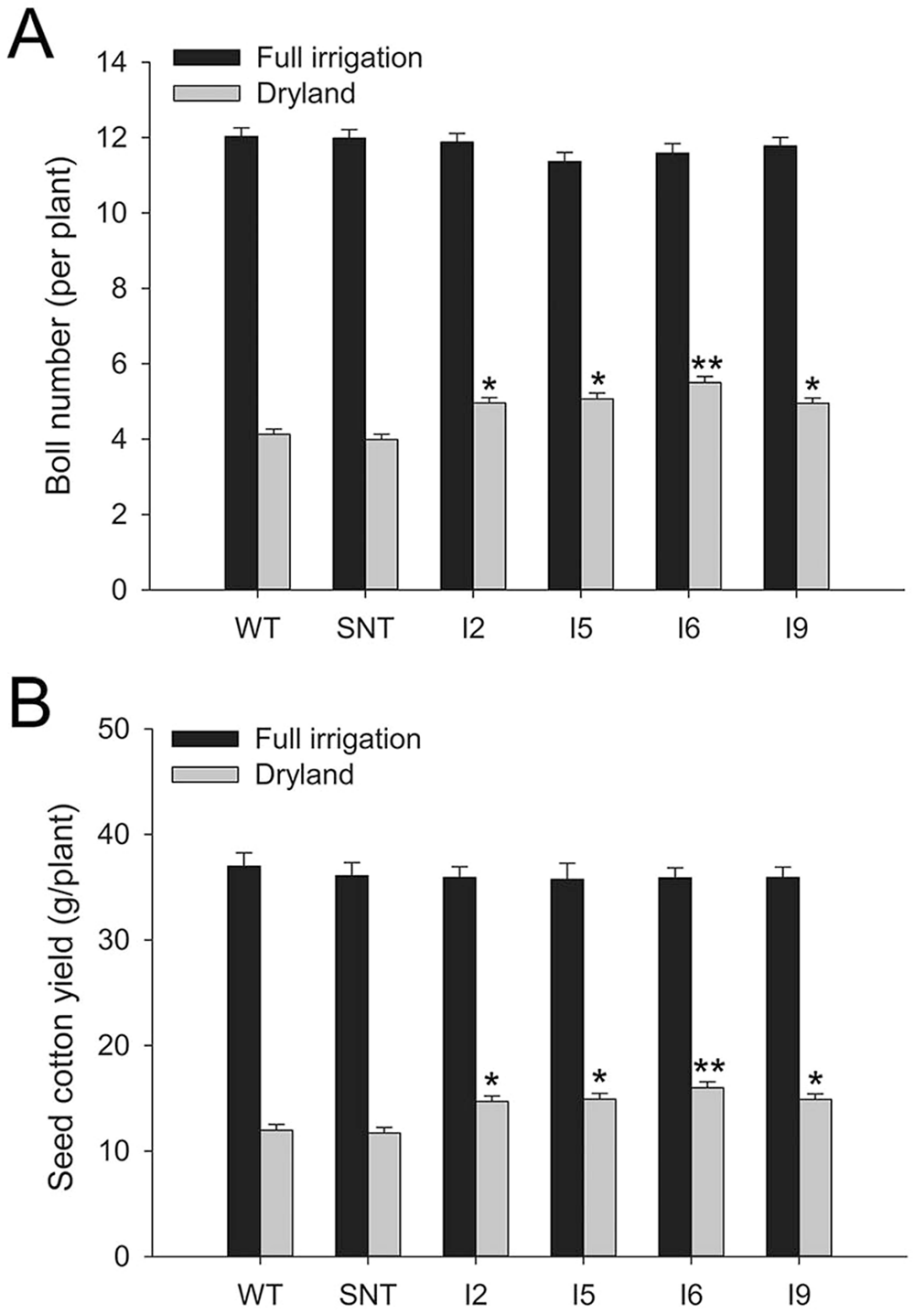
Boll number and seed cotton yields of cotton lines under full irrigation and dryland conditions in 2011. WT, wild-type cotton (Coker 312); SNT, segregated non-transgenic cotton; IPT2 to IPT9, four independent *IPT*-transgenic cotton lines. Black bar, full irrigation condition; grey bar, dryland condition. *Statistically significant at the <0.05 level; **statistically significant at the < 0.01 level.

We previously reported that regulated overexpression of *IPT* in cotton increased drought tolerance in growth chamber and greenhouse conditions^21^. To evaluate the performance of *IPT*-transgenic cotton in field conditions, the four independent *IPT*-transgenic cotton lines, IPT2, IPT5, IPT6 and IPT9, along with the control lines, wild-type Coker 312 (WT) and segregated non-transgenic (SNT), were grown at the Experimental Farm of USDA-ARS Cropping Systems Research Laboratory in Lubbock County, Texas in 2010 and 2011. The weather information during these two growth seasons are provided in Supplemental Table 1. In 2010, the experiment was conducted on a small scale of three duplicates with a total of 90 plants per line. Results from 2010 indicated that *IPT*-transgenic cotton lines had higher photosynthetic rates than their WT and SNT counterparts under dryland conditions (Fig. 1A). Consequently, these *IPT*- transgenic cotton lines produced more bolls and an average higher seed cotton yield of 44% than control lines under this field condition (Fig. 1B,C). There were no significant differences in the number of bolls and yield of seed cotton between control and *IPT*- transgenic cotton lines when they were grown under full irrigation conditions. Results from 2011 also showed that *IPT*- transgenic cotton lines performed better than control lines for boll number and seed cotton yield. Under dryland conditions, on average, *IPT*-transgenic cotton lines produced 27% higher seed cotton yield than control lines (Fig. 2). Because 2011 was the driest and hottest season in the last one hundred years in Lubbock (Supplemental Table 1), seed cotton yield from full irrigated plots was also substantially reduced (comparing Figs 1 and 2).

### There were no statistical differences in fiber quality traits between *IPT*-transgenic cotton and control cotton lines under full irrigation and dryland conditions in the field

**Figure 3:**
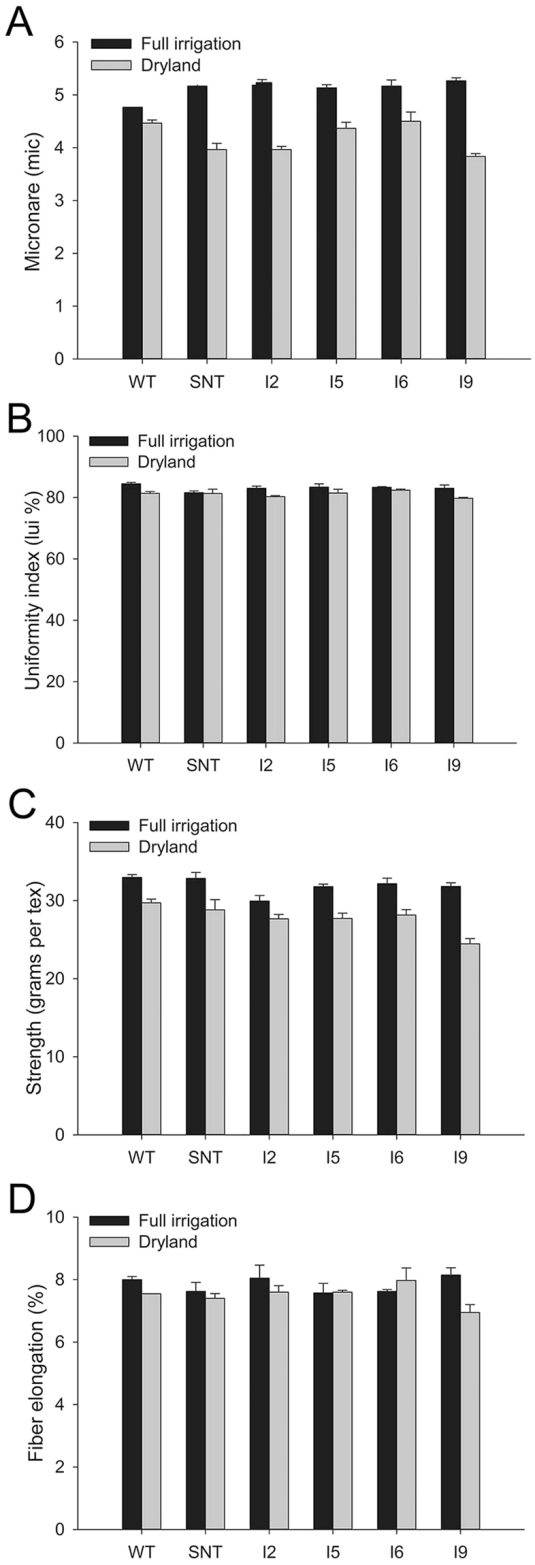
Fiber quality analyses for cotton lines grown under full irrigation and dryland conditions in 2011. WT, wild-type cotton (Coker 312); SNT, segregated non-transgenic cotton; IPT2 to IPT9, four independent *IPT*-transgenic cotton lines.

The qualities of fiber harvested from cotton plants grown under full irrigation and dryland conditions in 2011 were analyzed using high volume instrument (HVI) testing. When grown under full irrigation conditions, the micronaire of cotton fiber from WT was around 4.8, which was slightly lower than those from SNT and transgenic lines (Fig. 3A). However, there were no statistical significant differences (α = 0.05) between SNT and transgenic cotton lines. Under dryland conditions, the micronaire of fiber from WT and SNT were 4.4 and 3.9, respectively. The average micronaire of the four independent *IPT*-transgenic cotton lines ranged from 3.9 to 4.5 (Fig. 3). There were no significant differences (α = 0.05) between non-transgenic and transgenic lines. Similarly, under full irrigation conditions, there were no significant differences (α = 0.05) in micronaire between control and transgenic lines (Fig. 3). In addition, three fiber traits, uniformity, strength, and length, were also analyzed, and again there were no significant differences (α = 0.05) observed between control cotton and transgenic lines (Fig. 3).

### Different results were obtained from the field study at Maricopa of Arizona

**Figure 4:**
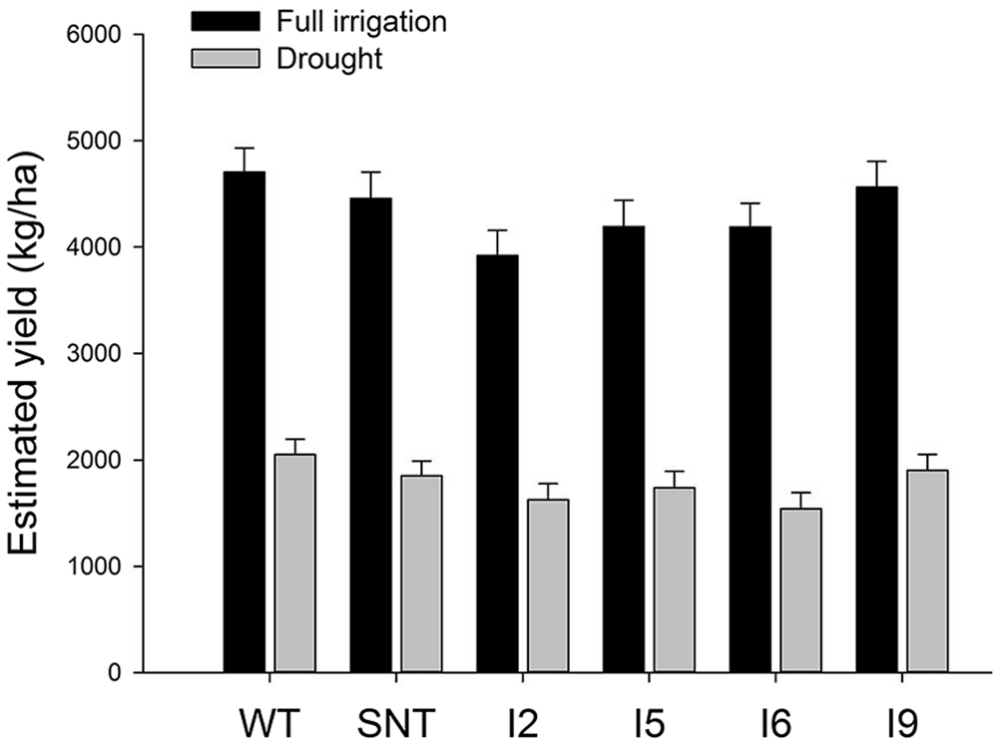
Seed cotton yield under full irrigation and water deficit conditions at Maricopa, Arizona in 2011. WT, wild-type cotton (Coker 312); SNT, segregated non-transgenic cotton; IPT2 to IPT9, four independent *IPT*-transgenic cotton lines.

The results from laboratory experiments^21^ and from field experiments in Lubbock, Texas, in 2010, suggested that *IPT* could be used for improving drought tolerance in cotton. To test if similar results could be obtained at another location with contrasting environmental and crop management conditions, in 2011, we conducted a field experiment at Maricopa, Arizona, one of the driest and hottest agricultural production sites in North America. For the water deficit plots, a coincident full irrigation schedule was applied until all plots were at first flower. Thereafter, irrigation of water deficit plots was conducted at ~50% of the full irrigation amount until the end of the experiment. When analyzing seed cotton yield at the end of the growing season, we found that *IPT*-transgenic cotton plants did not outperform control lines (WT and SNT) under both full irrigation or water deficit conditions (Fig. 4). The water deficit condition imposed at first flowering did cause a decrease in final yield for all lines, but it failed to cause statistically significant (α = 0.05) yield differences between control and transgenic plants. In fact, all transgenic lines produced lower yields in comparison to WT lines, although the differences were not statistically significant (α = 0.05) (Fig. 4).

### New experimental designs to explain the different field results

**Figure 5:**
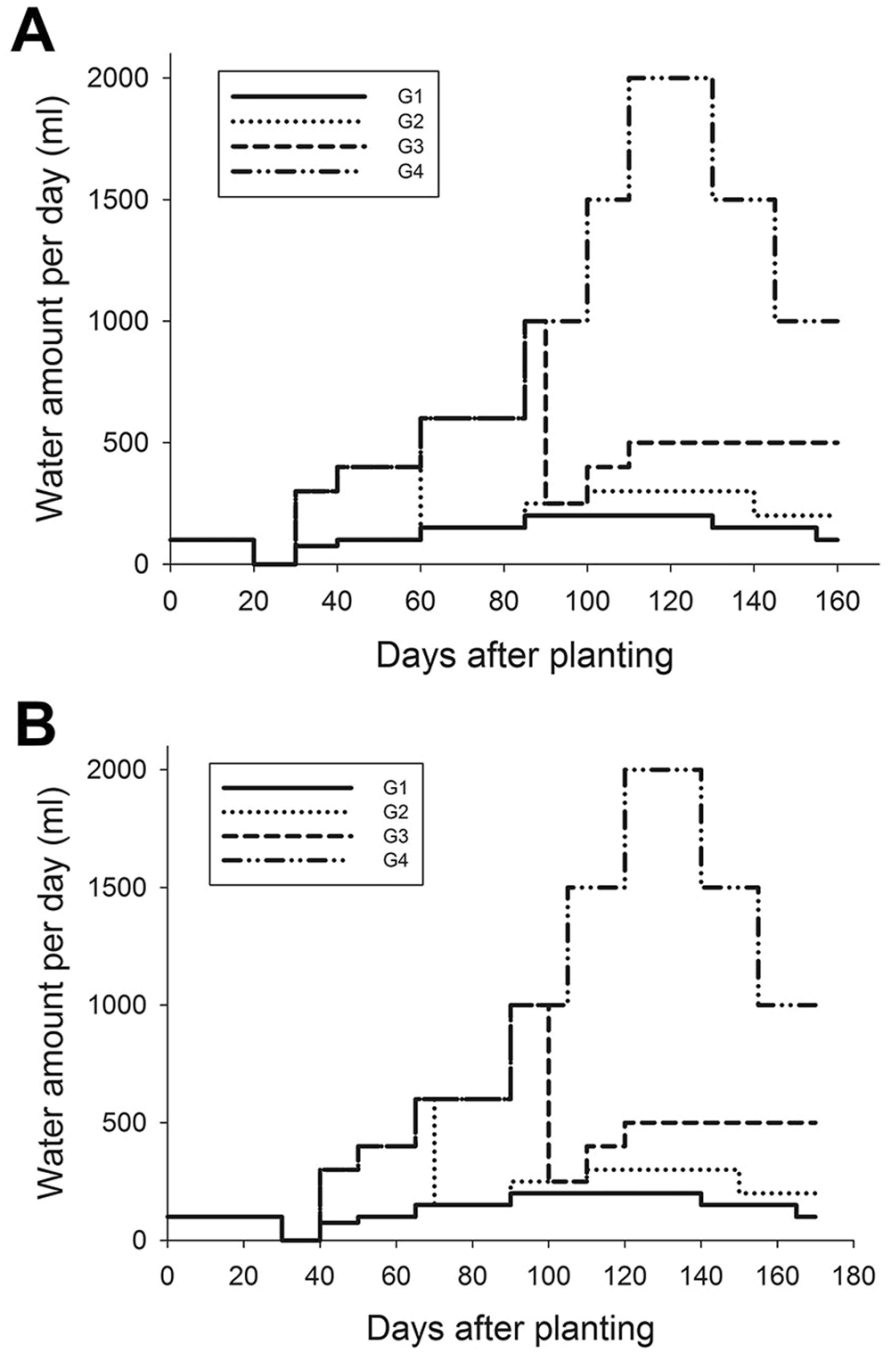
Experimental design for differential irrigation experiments in greenhouse. G1, G2, G3, and G4, four groups of plants with different irrigation amount and timing.

In contrast to Texas, the yield advantage of *IPT*-transgenic cotton under water deficit conditions was not observed in Arizona. The environmental conditions in Maricopa, Arizona cannot support dryland agriculture due to low precipitation and high temperatures in the summer (see the summer weather condition in Maricopa in 2011, Supplemental Table 2), therefore we had to irrigate cotton for both full irrigation and water deficit treatments. We hypothesized that the timing of water deficit stress might make a difference for different cotton plants, given that the water deficit stress was imposed several days after first flower in the Arizona field trial. Therefore, we designed two new experiments to test this hypothesis. In the first experiment, we applied differential irrigation on day 30 after planting (Fig. 5A), and we divided cotton plants into four groups, G1, G2, G3, and G4. On day 30, the G1 group was irrigated with ¼ of the water that was used for the other 3 groups until the day 60. By then the irrigation for the G2 group was dropped to the amount identical to G1 group (i.e., ¼ of the water used for G3 and G4 groups) (Fig. 5A). By day 90, the irrigation for the G3 group was dropped to the amount identical to the G2 group (i.e. ¼ of the water used for the G4 group). The G4 group never faced water deficit during the whole growth season, therefore it served as the fully irrigated control group. For the second experiment, the differential irrigation was applied on day 40 after planting and the irrigation schedule was similar to the first experiment, except the time for water deficit treatment was delayed by 10 days (Fig. 5B).

### The timing of water-deficit stress is critical for *IPT*-transgenic plants to show higher yield under water-deficit stress conditions

**Figure 6:**
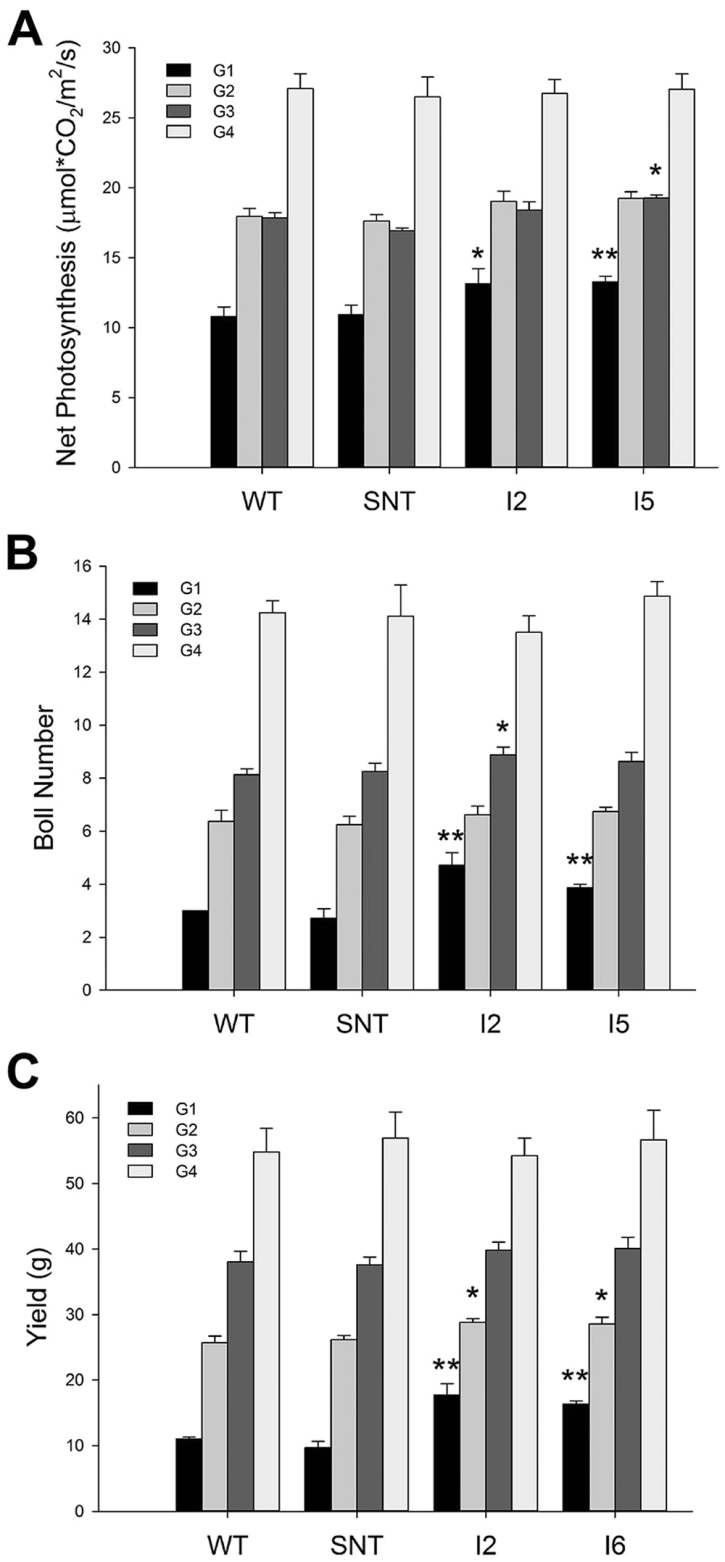
Photosynthetic rate, boll number and cotton seed yield of cotton lines under differential irrigation conditions in greenhouse. WT, wild-type cotton (Coker 312); SNT, segregated non-transgenic cotton; IPT2 to IPT9, four independent *IPT*-transgenic cotton lines.

We conducted photosynthetic analysis during the water-deficit treatments and analyzed final fiber yields for cotton plants that were differently treated at the end of experiments. For the first experiment, we could detect significant differences in photosynthetic rate, boll number, and fiber yield between control and *IPT*-transgenic lines in the G1 group (Fig. 6). These data indicate that when the differential irrigation was applied on day 30 after planting and continued to the end of growth season, *IPT*-transgenic lines outperformed control lines by producing 50% more seed fibers. If the differential irrigation was applied on day 60 after planting and continued to the end of growth season, *IPT*-transgenic lines had slightly higher but non-significant photosynthetic rates than control plants. Yet, *IPT*-transgenic lines still produced 5% higher seed fiber yields than control lines. When the differential irrigation was applied on day 90 after planting and continued to the end of growth season, one *IPT*-transgenic line still had a slightly higher photosynthetic rate, and another line produced slightly more bolls per plant, but there were no differences in seed fiber yields between control plants and *IPT*-transgenic lines.

**Figure 7:**
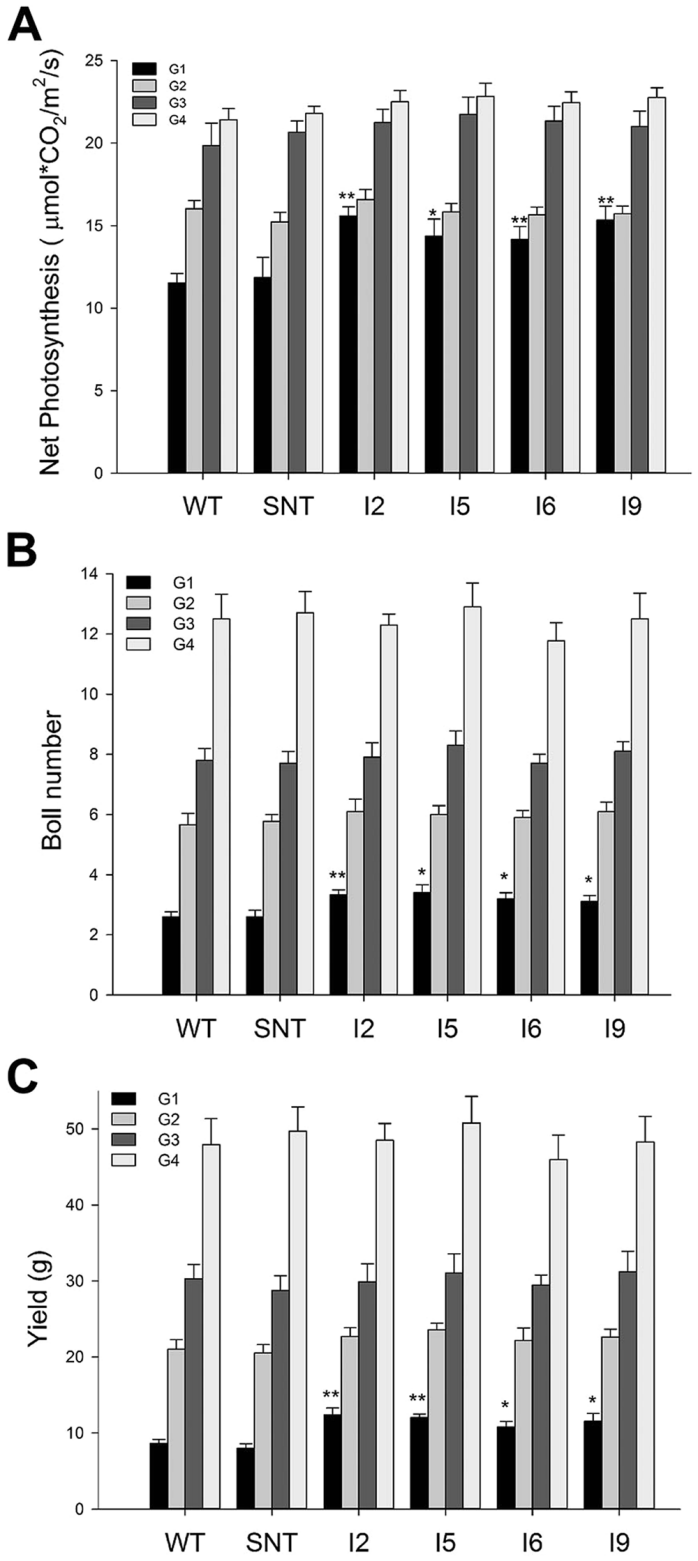
Photosynthetic rate, boll number, and cotton seed yield of cotton lines under differential irrigation conditions in greenhouse. WT, wild-type cotton (Coker 312); SNT, segregated non-transgenic cotton; IPT2 to IPT9, four independent *IPT*-transgenic cotton lines.

In the second experiment, we added two more transgenic lines in the experiment and we delayed the start of differential irrigation by 10 days. This time, we only detected significant differences in photosynthetic rate, boll number, and seed fiber yield between control and *IPT*-transgenic lines in the G1 group (Fig. 7). We did not detect any differences for the same three traits between control and *IPT*-transgenic lines in the other three differential irrigation groups, G2-G4. Our data indicated that the timing of the water deficit treatment is critical to the final yield differences, and after day 70, water deficit treatment will not cause any significant differences between control and *IPT*-transgenic lines.

## Discussion

In this study, we field-tested *IPT*-transgenic cotton plants at two sites, Texas and Arizona, and we obtained different results from these two sites. In Texas, we found that *IPT*-transgenic cotton lines outperformed control plants under dryland conditions in 2010 and 2011 (Figs 1 and 2). In contrast, we did not detect yield differences between *IPT*-transgenic cotton plants and control plants under irrigated and water deficit conditions in the Arizona trial (Fig. 4). To explain the reason for obtaining different results in Arizona, we designed new experiments to test our hypothesis that the timing of water deficit stress is critical for *IPT*-transgenic cotton lines to show its advantage over control lines. Based on our greenhouse experiments, we found that there seems to be a short window, i.e. 60 to 70 days after planting, during which time the water deficit treatment to cotton plants would make a difference in yield between *IPT*-transgenic cotton and control lines (WT and SNT). If the water deficit treatment starts before day 60, we would detect yield differences; however, if applied after day 70, we would not be able to detect yield differences (Figs 6 and 7). The yields from plants treated with water deficit on day 70 were less than half of those fully irrigated plants, which was similar to the yield differences obtained from the Arizona field trial (Fig. 4). The cotton plants grown in the Arizona trial resembled the G2 plants (70 days after planting) of our second greenhouse experiments (Fig. 7), and no yield differences were found between *IPT*-transgenic cotton plants and control plants. The 10-day difference between these two experiments are probably caused by the following two reasons: (1) the temperature in Arizona was much higher than that in greenhouse, which caused plants grown in Arizona aged earlier than plants grown in greenhouse or the 60 days old plants grown in Arizona were developmentally similar to the 70 days old plants grown in greenhouse; (2) the withdrawal of furrow irrigation on day 60 likely did not cause water deficit stress until the day 70 given the high saturation and slow drying rate of the soil.

The yield differences between *IPT*-transgenic cotton and control cotton lines were due to more bolls being retained in *IPT*-transgenic cotton under water deficit treatment. It appears that *IPT*-transgenic cotton plants lose less bolls when the water deficit stress is applied early (before day 60 after planting in greenhouse); however, if the water deficit stress is applied late in cotton development, e.g. 70 days after planting, *IPT*-transgenic cotton and wild-type cotton display no difference in boll retention. Our experiments indicate that the *IPT* gene can improve cotton productivity by reducing yield loss only if water deficit stress is constantly present or occurs early in cotton growth and development, roughly before two months into cotton’s growth. This period is around the transition from vegetative phase to reproductive phase in cotton’s development. If water deficit stress occurs late, i.e. after first flowering, then the benefits of expression of *IPT* is lost. Our experiments in greenhouse indicated that *IPT*-transgenic cotton plants developed larger root systems under reduced irrigation condition early on in cotton’s growth and development^21^, therefore by the time plants started to flower, *IPT-*transgenic plants already had higher capacity to absorb more water, thereby better able to adapt to water-deficit stress. Plants are most sensitive to heat stress during flowering, as pollination process is heat sensitive. If water-deficit stress occurs too late, for examples, 70 days after planting in our greenhouse experiments or two months after planting in our Arizona field trails, *IPT*-transgenic plants did not have the opportunity to develop a larger root system, we, of course, should not expect yield difference. This might be the reason why we saw yield differences in trials conducted in Lubbock, not yield differences in trials conducted in Arizona. Our research could serve as a cautionary note for efforts in using *IPT* to improve drought tolerance in other crops

Many genes have been used in efforts to improve drought tolerance in plants over the last 20 years, yet very few were successfully employed in real world conditions^5,6^. The high failure rate in commercializing the genes that were promising in laboratory experiments is likely due to negative results from the field trials. To be useful in agriculture, a gene should withstand vigorous tests both in the laboratory and field. Under well irrigated conditions, *IPT*-transgenic plants do not show any yield loss in comparison to wild-type plants^12,21^, which indicates that regulated expression of *IPT* does not lead to yield penalty. In water-limited environments, e.g. semiarid agricultural conditions, *IPT*-transgenic crops will likely show its advantage. In this regard, the *IPT* gene still has value in agricultural biotechnology.

In 2010, *IPT*-transgenic cotton lines displayed higher photosynthetic rates than control lines under dryland conditions in the field (Fig. 1). On average, *IPT*-transgenic cotton plants produced 44% more seed cotton than control plants under dryland conditions (Fig. 1). In 2011, these *IPT*-transgenic cotton lines again outperformed control plants, albeit at lower levels when compared to the 2010 results. On average, transgenic plants produced 27% more seed cotton than control plants under dryland conditions (Fig. 2). Under full irrigation, however, there were no significant differences between *IPT*-transgenic cotton and control lines. The performance of transgenic lines in 2010 was better than in 2011, and the likely explanation was due to the dramatic differences in weather conditions. In 2010, there was 36.99 cm of precipitation during the cotton growing season (June to October, Supplemental Table 1), whereas in 2011, the Lubbock area experienced a record drought and high summer temperatures (Supplemental Table 1). For example, the rainfall from June to October in 2011 was only 7.57 cm, which was the lowest in the 75 years recorded history of Lubbock (Supplemental Table 1). During this time, there were 49 consecutive days with a daytime temperature over 38 °C, and the soil temperature reached as high as 65 °C in July. This led to a yield in the dryland condition that was only 1/3 of the yield obtained under dryland condition in 2010 (Fig. 2). It appears that the differences in the performance of *IPT*-transgenic cotton in the field between 2010 and 2011 were caused by the combined severe heat stress and drought stress. We observed that during the extended period of drought, control plants dropped more flowers in comparing to the *IPT*-expressing plants in both 2010 and 2011 growth seasons. Drought induced up-regulation of isopentenyltransferase, and consequent up-regulation of cytokinin, may be the reason for the delayed senescence and abscission of flowers during this extended period of drought. Also, cytokinin mediated protection of photosynthetic machinery that leads to higher photosynthetic rates during the recovery phase could have contributed to the relatively higher fiber yield in the end. Based on the data from greenhouse, growth chamber, and field, it is evident that the *IPT*-transgenic cotton is indeed more drought tolerant than controls under water deficit conditions, especially when the water deficit stress occurs early in cotton’s growth and development.

## Materials and Methods

### Drought treatment in greenhouse

WT, SNT and 2 and 4 independent *IPT*-transgenic lines from our previous study^21^ (2 lines were used in the experimental design I and 4 lines were used in the experimental design II) were sown in 3 gallon pots (11.36 L) in potting mixture. Plants were allowed to germinate and establish for a period of 20 days, during which the pots were irrigated with 100 ml of water daily. After day 20, water was withheld for all pots for 10 days before differential irrigations were started. In the first experiment, differential irrigation was started on day 30 (Fig. 5A), where the G1 group was irrigated with ¼ of the water that was used for G2, G3 and G4 groups until the day 60, then the irrigation for the G2 group was dropped to the amount identical to G1 group. The differential irrigations for G1/G2 and G3/G4 were maintained at 4-fold (Fig. 5A). On day 90, the irrigation for G3 plants was dropped to the amount identical to the G2 group (i.e. ¼ of the water used for G4 plants). The irrigation for G4 plants was increased as plants increased in size, but it was always maintained 4-fold higher than the next group of plants (i.e. G3). In the second differential irrigation experiment, the time for differential irrigation was applied on day 40 instead of day 30. All treatments were continued until boll development and maturation. During the drought treatment, photosynthetic rates were measured at each treatment period. At the end of the treatments, bolls per plant and fiber yield per plant were analyzed. Fourteen biological replicates for each line were used in each differential irrigation experiments.

### Field studies in Texas and Arizona

WT, SNT and four independent *IPT*-transgenic cotton lines were grown in the Experimental Farm of the USDA-ARS Cropping Systems Research Laboratory in Lubbock, Texas during the cotton growth seasons of 2010 and 2011. During the 2010 growth season duplicates with a total of 90 plants per line were sown in paired rows. Controls and *IPT*-transgenic lines were grown under full irrigation (38 mm per week), low irrigation (19 mm per week), and dryland and conditions. The border rows were planted with cotton having colored leaves so that they could be spotted easily. At the end of the growth season, the boll number per plant and seed cotton yield per plant were measured.

The WT, SNT and four independent *IPT*-transgenic cotton lines were evaluated at the Maricopa Agricultural Center (MAC) of the University of Arizona, located in Maricopa, AZ (33°04′37″ N, 111°58′26″ W, elevation 358 m) in 2011. The set of six experimental lines was evaluated under full irrigation and water deficit conditions. We planted the field trial on day 123 (Julian calendar) in 2011. The experimental field trial was arranged as a randomized complete block design with four replications for each irrigation regime. To reduce edge effects, several border rows of a commercial upland cotton cultivar were planted on all sides of each set of four replicates. Experimental units were one-row plots, 10.67 m in length, with a 3.05 m alley at the end of each plot. Plots had an average density of ∼6.2 plants m^−2^ and a spacing between rows of 1.02 m. The soil type is a Casa Grande sandy loam (fine-loamy, mixed, superactive, hyperthermic Typic Natrargids). Scheduling of furrow irrigations was performed using a daily soil water balance model calculated for the cotton root zone following the method previously described^22^. Irrigations to the full irrigation plots were applied to refill the root zone water content to field capacity at approximately 35% soil water depletion. Starting mid-July, the water deficit plots received one-half of the irrigation amounts applied to the full irrigation plots. The imposition of the water deficit occurred when all experimental plots were at first flower (i.e., a plot had at least one plant with a visible flower). All experimental plots were mechanically harvested using a one-row harvester on day 306 (Julian calendar) to measure seed cotton yield (kg ha^−1^).

### Measurement of leaf gas exchange and photosynthetic rate

To assess the photosynthetic performance of the WT, SNT and *IPT*-transgenic cotton plants under full irrigation, reduced irrigation, and dryland conditions in the field, gas exchange measurements were taken with a portable photosynthesis system Li-6400 (LI-COR Inc., Lincoln, NE). Readings were taken on the 3rd fully expanded intact leaves of plants that were 60 days into the treatment. Environmental parameters in the measurement chamber were set at temperature 25 °C, and air flow rate was 500 μmol s^−1^ and light intensity of 1500 μmol m^−2^ s^−1^. Net photosynthetic rate and transpiration were assessed at a CO_2_ concentration of 400 μmol/mol. The instrument was allowed to warm and stabilize as per the manufacturer’s instructions. Steady state levels of reference CO_2_ and reference H_2_O were observed before taking measurements. The sample and the reference IRGAs (infra-red gas analyzers) were matched manually before measurements. Five readings were logged for each sample.

### Statistical analysis

Student *t*-test considering one tailed unequal variance was performed to compare the performance of WT, SNT and *IPT*-transgenic lines. All *P* values were from comparison between control (WT and SNT) and transgenic lines. Statistical analysis was performed using Microsoft^®^ Office Excel 2007.

Statistical significance of variation for seed cotton yield data collected in Arizona was tested by analysis of variance (ANOVA). Briefly, a mixed linear model was fitted with PROC MIXED in SAS ver. 9.3 (SAS Institute). The fitted model had the plot raw value of seed cotton yield as the dependent variable. For the explanatory variables, irrigation treatment, line, and their interaction were fitted as fixed effects. Replicate nested within irrigation treatment and row of the physical field grid layout were fitted as random effects. Likelihood ratio tests were conducted to remove all terms fitted as random effects from the model that were not significant at α = 0.05. Cotton seed yield values presented in the manuscript are least square means (LSM) and their standard errors for each line by irrigation treatment. We used the LSMEANS statement in SAS to conduct pairwise comparisons between control and transgenic lines with a *t* test of the null hypothesis that the difference was zero. Statistical significance for a non-zero value was declared at α = 0.05. The Tukey–Kramer method was used for post hoc multiple pairwise comparisons between least square means (LSM) of the levels for each fixed effect.

## Electronic supplementary material

Supplemental Materials
