# Supplementary material for: The yield difference between wild-type cotton and transgenic cotton that expresses IPT depends on when water-deficit stress is applied
Source: Sci Rep. 2018 Feb 7;8:2538. doi: 10.1038/s41598-018-20944-7 (PMC5803251; doi:10.1038/s41598-018-20944-7)
Supplement: Supplementary file 1 — Supplemental Materials [file 41598_2018_20944_MOESM1_ESM.pdf]

Supplemental Materials

The yield difference between wild-type cotton and transgenic cotton that expresses *IPT* depends on when water-deficit stress is applied

Xunlu Zhu[1, 6], Li Sun[1, 6], Sundaram Kuppu[1, 6], Rongbin Hu[1], Neelam Mishra[1], Jennifer Smith[1], Nardana Esmaeili[1], Maheshika Herath[1], Michael A. Gore[2, 5], Paxton Payton[3, *], Guoxin Shen[4, *], Hong Zhang[1, *]

[1] Department of Biological Sciences, Texas Tech University, Lubbock, TX 79409, USA

[2] USDA-ARS, Arid-Land Agricultural Research Center, Maricopa, AZ 85239, USA

[3] USDA-ARS, Cropping Systems Research Laboratory, Lubbock, TX 79415, USA

[4] Zhejiang Academy of Agricultural Sciences, Hangzhou, Zhejiang Province, China

[5] Current address: Plant Breeding and Genetics Section, School of Integrative Plant Science, Cornell University, Ithaca, NY 14853, USA

[6] Co-first authors

*Co-corresponding author: Hong Zhang (phone: 806-834-1579; fax: 806-742-2963; e-mail, hong.zhang@ttu.edu); Paxton Payton (paxton.payton@ars.usda.gov); Guoxin Shen (e-mail: guoxin.shen@gmail.com).

Supplemental Tables

Table 1. Average monthly high and low temperatures and total precipitation for Lubbock, TX, USA in 2010 and 2011. Source: National Weather Service Weather Forecast Office, Lubbock, TX (http://www.srh.noaa.gov/lub).

Year 2010

**Lubbock**	**Average High Temperature** (°C)	**Average Low Temperature** (°C)	**Precipitation** (cm)
January	11.78	-3.37	3.58
February	9.89	-1.83	4.52
March	18.83	2.61	7.24
April	23.11	9.06	11.81
May	28.06	13.61	2.90
June	34.39	20.00	6.48
July	30.28	20.11	18.14
August	34.00	19.61	3.38
September	31.06	16.56	2.36
October	26.33	8.83	6.63
November	19.39	1.50	0.18
December	15.89	-1.00	0.00
**Annual**			**67.22**

Year 2011

**Lubbock**	**Average High Temperature** (°C)	**Average Low Temperature** (°C)	**Precipitation** (cm)
January	13.89	4.44	0.15
February	14.44	-3.89	1.09
March	22.78	3.33	0.89
April	36.11	16.66	0.00
May	30.56	12.77	0.66
June	38.33	21.66	0.00
July	37.22	22.77	0.13
August	37.22	22.77	0.86
September	30.00	13.88	3.18
October	25.00	8.88	3.40
November	17.78	10.55	0.66
December	7.78	2.77	3.86
**Annual**			**14.88**

Table 2. Average monthly high and low temperatures and total precipitation for Phoenix, Arizona, USA in 2011. Source: National Weather Service Weather Forecast Office, Phoenix, AZ (http://w2.weather.gov/climate/xmacis.php?wfo=psr).

Year 2011 **Phoenix**	**Average High Temperature** (°C)	**Average Low Temperature** (°C)	**Precipitation** (cm)
January	24.4	-1.1	0.10
February	27.2	-1.1	1.67
March	33.8	8.3	0.15
April	37.7	6.6	0.68
May	38.3	12.7	0.02
June	46.1	18.8	0.00
July	47.7	23.3	3.58
August	47.2	27.2	0.43
September	44.4	22.2	0.05
October	37.7	13.8	0.28
November	31.1	7.2	2.05
December	23.8	2.2	2.79
**Annual**			**11.83**
